# Lipoprotein(a)-dependent cardiovascular risk in different populations: are thresholds the same?

**DOI:** 10.1080/07853890.2026.2727235

**Published:** 2026-10-10

**Authors:** Brett S. Mansfield, Frederick J. Raal, Petri T. Kovanen, Alpo Vuorio

**Affiliations:** a Carbohydrate and Lipid Metabolism Research Unit, Department of Internal Medicine, Faculty of Health Sciences, University of the Witwatersrand, Johannesburg, South Africa; b Cardiovascular Research Laboratory, Wihuri Research Institute, Helsinki, Finland; c Mehiläinen Aviapolis Health Centre, Vantaa, Finland; d Department of Forensic Medicine, University of Helsinki, Helsinki, Finland

**Keywords:** Ancestry, atheroclerotic vascular disease, cardiovascular risk, lipoprotein(a), threshold

## Abstract

**Background:**

Lipoprotein(a) [Lp(a)] is a genetically determined, independent and causal risk factor for atherosclerotic cardiovascular disease (ASCVD) and aortic stenosis. Large prospective cohort studies together with Mendelian Randomization studies have established its role in cardiovascular risk which has led to the introduction of screening and the development of Lp(a)-lowering therapies. However, interpretation of Lp(a) results in clinical practice remains a challenge, particularly across populations with significantly different baseline levels.

**Discussion:**

Lp(a) levels vary according to ancestry. People of African ancestry having the highest median Lp(a) levels, followed by people from South Asia, East Asia and Europe, respectively. However, despite these differences, current Lp(a) risk thresholds are derived from populations of predominantly European ancestry and broadly applied across underrepresented population groups.

Available evidence from multi-ancestry cohort studies have shown that while proportional risk for ASCVD may be similar across ancestries, the absolute risk associated with specific thresholds may differ. The application of a universal threshold may lead to over-classification of risk in some populations, resulting in unnecessary edicalization and strain on healthcare systems. Conversely, some populations may be under-classified by a universal threshold potentially resulting in avoidable ASCVD events.

**Conclusion:**

This commentary explores the epidemiological, biological and methodological factors underlying variability in Lp(a) levels and their association with ASCVD across populations. We discuss the advantages and limitations of applying ancestry-specific Lp(a) risk thresholds. Lp(a) measurements in clinical practice should be interpreted in within a broader clinical and population context rather than in isolation.

## KEY MESSAGES

- Lipoprotein(a) levels vary markedly between ancestral groups.
- Current lipoprotein(a) cardiovascular risk thresholds are skewed towards studies involving people of European ancestry.
- There is a need for future studies to ensure adequate diversity which would allow for broad conclusions and greater applicability of study results.

## GRAPHICAL ABSTRACT

**Figure:**
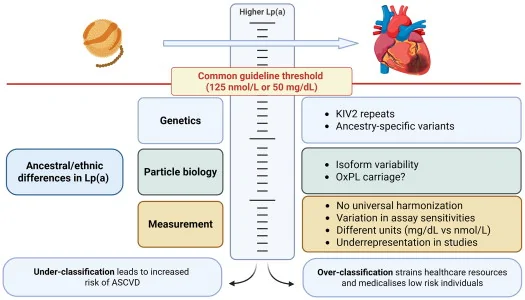

Graphical abstract provided as a separate file. (Graphical abstract created in https://BioRender.com)

## Introduction

Over the last few decades, large prospective epidemiological studies together with Mendelian randomization studies have firmly established lipoprotein(a) [Lp(a)] as a causal risk factor for atherosclerotic cardiovascular disease (ASCVD) and aortic stenosis (AS) independent of the traditional cardiovascular risk factors [1–9]. Despite this, the interpretation of Lp(a) levels in clinical practice remains challenging, particularly across populations with significantly different baseline levels [2].

Lp(a) is an LDL-like particle consisting of an apolipoproteinB (apoB) particle covalently bound to an apolipoprotein(a) tail. Lp(a) represents a small fraction of the circulating atherogenic apoB containing lipoproteins, but, in European populations, it has been shown to carry a five to six fold greater atherogenicity compared to LDL on a per particle basis [10,11].

Lp(a) is 85–90% genetically determined and levels are not meaningfully influenced by diet, lifestyle or currently available lipid-lowering therapies like statins and ezetimibe [1,12]. At present, no specific therapies (apart from apheresis) are available for individuals with an elevated Lp(a). Cardiovascular outcome trials are currently underway to test whether lowering Lp(a) will reduce ASCVD events. Drugs currently in development include an antisense oligonucleotide (pelacarsen), small interfering RNA (olpasiran, zerlasiran and lepodisiran) and an oral small molecule (muvalaplin) [13–17]. Despite the lack of approved therapies, the cardiovascular risk associated with elevated Lp(a) is still actionable. Current guidelines recommend strict control of all modifiable risk factors to minimise cardiovascular risk [1,18,19].

This commentary will describe the differences seen between population groups and explore the evidence supporting the current Lp(a) risk thresholds. The advantages and disadvantages of ancestry-specific risk thresholds for Lp(a) will be discussed.

## Discussion

### Differences across population groups

Median Lp(a) levels and the distribution of Lp(a) vary significantly across people of different ancestry [2,20]. Those of African descent have median Lp(a) levels 3–4 times that of their European counterparts [2]. South Asians have median Lp(a) levels which fall between that of Africans and Europeans, while those of East Asian ancestry have the lowest Lp(a) levels [2].

These population-level differences are primarily genetically driven. The *LPA* gene, located on chromosome 6, contributes substantially to this variation in Lp(a) levels. The major determining factor are the size differences in *LPA* gene isoforms. Isoform sizes are the result of the number of kringle 4 type 2 (KIV_2_) repeats located within the *LPA* gene [21]. The number of KIV_2_ repeats may range between 1 and >40 repeats. The plasma concentration of Lp(a) is inversely correlated with isoform size. Simply, small isoforms are produced rapidly within the liver, leading to higher Lp(a) levels, while larger isoforms take longer to produce and lead to lower Lp(a) levels [21,22]. People of African descent, however, have moderately sized isoforms yet the highest Lp(a) levels so there must be other factors involved [22].

In addition to isoform size, numerous single nucleotide polymorphisms (SNPs) within the *LPA* gene are associated with higher or lower Lp(a) levels and may contribute further to this variability [23]. The prevalence of SNPs varies by ancestry. In European cohorts, rs3798220 and rs10455872 are associated with small isoforms and elevated Lp(a) [4]. In Hispanic Americans, rs3798220 is associated with large isoforms and low Lp(a) levels [21]. However, rs3798220 was not associated with elevated Lp(a) in Asians [24]. These particular SNPs are absent in people of African ancestry who have a markedly different spectrum of SNPs [24,25]. Some SNPs are associated with lower Lp(a) levels and a reduced risk of ASCVD [23]. Fine mapping of the *LPA* locus in the UK Biobank showed that loss-of-function variants are less common in people of African ancestry, while the Lp(a)-raising 5′UTR variant rs1800769 is more common [26]. Consistent with this, for a given number of KIV-2 repeats, allele-associated Lp(a) concentrations are higher in African than European populations [27,28]. Importantly, variation does not only occur between major geographic regions but also between ancestral groups within regions [29,30]. Between-population differences therefore reflect regulation of hepatic apo(a) production rather than isoform size alone. The mechanisms underlying the atherogenicity of Lp(a) are not well understood. While Lp(a) cholesterol content has been proposed as a contributor, the greater per-particle atherogenicity of Lp(a) compared with LDL-C suggests this cannot be the only mechanism [10]. The cholesterol content of Lp(a) has historically been reported as approximately 30% of the mass of Lp(a) [31]. However, a novel assay has shown considerable interindividual variability in Lp(a) cholesterol content of 6–57% relative to Lp(a) mass [32]. Whether, and how, this contributes to atherogenicity is not well understood. Furthermore, whether cholesterol content of Lp(a) varies substantially by ancestry is not known and warrants further study.

Beyond concentration alone, qualitative differences in Lp(a) particles may modify cardiovascular risk. Lp(a) particles are the principal carriers of oxidized phospholipids (OxPL) which are thought to be a major contributor to Lp(a) atherogenicity [33,34]. OxPL content varies by ancestry and apo(a) isoform size, both of which contribute to inter-individual variability [22,33]. OxPL is inversely associated with isoform size with small apo(a) isoforms exhibiting a broader range of OxPL content while larger apo(a) isoforms have a greater variability in Lp(a) concentrations but a narrower range of OxPL content [33]. If OxPL content per particle varies with isoform size, then thresholds based on Lp(a) mass may not capture equivalent atherogenic risk across populations with differing isoform distributions.

### Current guideline thresholds for Lp(a)

Lp(a) levels represent a continuum of cardiovascular risk, but thresholds are necessary and important markers of action and intervention for clinicians performing ASCVD risk assessments. A threshold is a decision rule which typically converts a continuous variable into an action (whether that be screening, treating and/or intervening). This manner of risk stratification is considered beneficial if those actions result in benefits that outweigh the cost or potential harms.

Guidelines differ with respect to actionable Lp(a) thresholds (see Table 1). The European Atherosclerosis Society (EAS) and European Society of Cardiology (ESC) recommend that an Lp(a) of ≥50 mg/dL (or ≥105 nmol/L) be considered as a cardiovascular risk-enhancing factor for all people [1,18]. The Canadian Cardiovascular Society (CCS) and American College of Cardiology (ACC)/American Heart Association (AHA)/National Lipid Association (NLA) multi-society guideline also propose that Lp(a) ≥50 mg/dL (≥125 nmol/L) represents a threshold of increased risk [35,36].

**Table 1.** Lipoprotein(a) thresholds recommended by major societies.

Society guideline	Lp(a) threshold	Population from which threshold derived	Recommended clinical action
European Atherosclerosis Society/European Society of Cardiology [1,18]	≥50 mg/dL (≥105 nmol/L)	Predominantly European ancestry	One lifetime measurement in all adults. Treat as risk enhancing factor. Control all modifiable risk factors including more intensive LDL-C lowering.
American College of Cardiology/American Heart Association/National Lipid Association [36]	≥50 mg/dL (≥125 nmol/L)	Predominantly European ancestry	One lifetime measurement in all adults. Considered a risk enhancing factor. Supports early control of all modifiable cardiovascular risk factors. Cascade screening of first-degree relatives.
Canadian Cardiovascular Society [35]	≥50 mg/dL (≥100 nmol/L)	Predominantly European ancestry	Risk modifier which prompts intensification of lipid-lowering therapy in those at intermediate risk. Promotes screening of first-degree relatives.
HEART UK [37]	Minor: 32–90 nmol/L Moderate: 90–200 nmol/L High: 200–400 nmol/L Very high: >400 nmol/L	Copenhagen General Population study; European ancestry	Risk factor optimisation at all levels. Intensive LDL-C lowering and specialist referral for those in high and very high-risk categories.
Australian Atherosclerosis Society [38]	Low: <100 nmol/L (<40 mg/dL) Moderate: 100–200 nmol/L (40–90 mg/dL) High: 200–400 nmol/L (90–180 mg/dL) Very high: >400 nmol/L (>180 mg/dL)	80th, 80–95th, 95–99th and ≥99th percentile of population of European ancestry	Graded intensification of risk factor control. Promotes family screening for those at high and very high levels.
Asia Pacific Society of Cardiology [39]	≥50 mg/dL	Extrapolated from diaspora populations of Southeast Asian descent	Risk enhancing factor. Intensify treatment of modifiable risk factors.
Japan Atherosclerosis Society [41]	No threshold stipulated	Significant heterogeneity across Japanese studies precludes a threshold	Lp(a) acknowledged as a factor contributing to cardiovascular risk, but no actionable threshold given.
Chinese guideline for lipid management [43]	≥30 mg/dL	Chinese ancestry	Should be measured at least once in those at high risk. Risk-enhancing factor which prompts intensification of modifiable risk factors.
Lipid Association of India [44]	Risk modifier: 20–49 mg/dL High risk: ≥50 mg/dL	Indian and South Asian ancestry	Risk modifier which informs more aggressive LDL-C targets while high risk category supports intensive lipid-lowering therapy.
Expert consensus on Lp(a) in the Gulf countries [45]	Increased risk ≥62 nmol/L High risk: ≥105 nmol/L Very high risk: ≥175 nmol/L Apheresis considered: ≥200 nmol/L (in those at very high CV risk)	Derived from EAS/ESC guidelines. No threshold derived from Gulf populations.	One lifetime measurement prioritized in FH, premature ASCVD or aortic stenosis or family history. Referral threshold ≥62 nmol/L (with other risk factors). Aggressive LDL-C management if Lp(a) ≥105 nmol/L. CAC score or CIMT to refine risk. Reverse cascade screening of first-degree relatives.
Brazilian Society of Cardiology [46]	Elevated: ≥50 mg/dL (≥125 nmol/L) Extreme: ≥180 mg/dL (≥390 nmol/L)	European and North American populations	Measure once in all adults. Cardiovascular risk enhancer. Primary prevention: early lifestyle counselling, consideration of vascular imaging and early statin initiation. Secondary prevention: more intensive LDL-C lowering and aggressive risk factor control. Cascade screening of family members.
Sociedad Argentina de Lipidos (SAL) [47]	>100–125 nmol/L (≈ 50 mg/dL)	European derived	One lifetime measurement in all adults but focused on premature ASCVD, aortic stenosis, FH and statin non-response. Informs statin decisions in those at intermediate cardiovascular risk. Repeat measurement advised in CKD, cirrhosis, hepatitis, uncontrolled hypothyroidism, post-menopausal hormone replacement.
SA Heart / Lipid Association of South Africa [48]	>50 mg/dL (>125 nmol/L)	Predominantly European ancestry (adopted from EAS/ESC guidelines)	Screening not recommended for general population. Can be considered for patients falling on the margin of moderate and high risk, those with FH, premature ASCVD, family history of premature ASCVD and/or elevated Lp(a), recurrent cardiovascular events despite risk factor control and Framingham 10-year risk ≥15%.

ASCVD: atherosclerotic cardiovascular disease; FH: familial hypercholesterolaemia; CAC: coronary artery calcium; CIMT: carotid intima media thickness; CKD: chronic kidney disease.

Both the UK and Australia describe multiple thresholds of risk. A consensus statement from HEART UK lists 4 different Lp(a) categories to designate risk [37]. These include those at minor (32–90 nmol/L), moderate (90–200 nmol/L), high (200–400 nmol/L) and very high (>400 nmol/L) risk [37]. These thresholds were derived from the Copenhagen General Population Study [3].

The Australian Atherosclerosis Society also has 4 thresholds to describe Lp(a) risk [38]. Low (<100 nmol/L or 40 mg/dL), moderate (100–200 nmol/L or 40–90 mg/dL), high (200–400 nmol/L or 90–180 mg/dL) and very high risk (>400 nmol/L or >180 mg/dL) which are derived from the 80th, 80–95th, 95–99th and 99th percentiles from a European population [38].

The Asia Pacific Society of Cardiology acknowledged the lack of evidence regarding Lp(a) on a regional level but refer to currently available evidence on diaspora populations of South East Asian descent wherein an Lp(a) ≥50 mg/dL denotes risk [39,40]. The Japan Atherosclerosis Society (JAS) note the contributions of Lp(a) to risk of ASCVD but do not stipulate a specific threshold of risk [41]. Significant heterogeneity in studies from Japan have made establishing a local risk threshold difficult [42]. In China, Lp(a) ≥30 mg/dL is deemed to be elevated [43]. The Lipid Association of India considers Lp(a) 20–49 mg/dL to be a risk modifier while levels ≥50 mg/dL are high risk [44].

### Epidemiological evidence supporting Lp(a) risk thresholds

Three important epidemiological studies, published in 2009, solidified Lp(a) as an independent risk factor for ASCVD and informed many of the aforementioned guidelines [3–5].

A pooled analysis of three Danish cohorts, specifically the Copenhagen General Population Study, the Copenhagen City Heart Study and the Copenhagen Ischemic Heart Disease Study established that genetically elevated Lp(a) was causal with respect to coronary heart disease [3]. In the Copenhagen City Heart Study, a 1.6-fold increase in risk for myocardial infarction was seen in people with an Lp(a) between 20–76 mg/dL compared to those with an Lp(a) < 5 mg/dL [3]. The often-quoted Lp(a) threshold of 50 mg/dL conforms to the 80th percentile in populations of European ancestry. Evidence for causality is significantly skewed towards people of European ancestry and there have been limited prospective or outcome-driven studies in African, South Asian and East Asian cohorts.

Secondly, the Emerging Risk Factors Collaboration produced a meta-analysis of 31 prospective studies which identified Lp(a) as an independent risk factor for fatal and non-fatal coronary heart disease as well as ischaemic stroke across the pooled cohort [5]. Black African participants had median Lp(a) levels that were 119% greater than their white counterparts. When analyzed by ancestral group, the incidence of coronary heart disease for every 1 standard deviation increase in Lp(a) was not significantly increased in those of African ancestry HR 1.05 (0.9–1.23) as it was in those of European ancestry HR 1.14 (1.09–1.19) [5]. More than 93% of all participants were reported as being of European ancestry while just 7% of the entire cohort were of African ancestry [5]. Too few people of South Asian or East Asian descent were reported to allow for any meaningful statistical analysis.

Finally, the Precocious Coronary Artery Disease (PROCARDIS) case-control cohort study recruited participants from four Western European countries (United Kingdom, Germany, Italy and Sweden) [4]. The *LPA* locus in this cohort was found to have the strongest genetic association with coronary artery disease compared to all other candidate genes [4].

Following these three landmark studies, further cohort studies comprising multi-ethnic groups were necessary to establish clear associations of Lp(a) and ASCVD in different population groups.

The Atherosclerosis Risk in Communities (ARIC) study included both African American and European American participants to investigate the risk of incident ASCVD over 20 years. Lp(a) was associated with incident ASCVD which was found to be similar between the different groups [49].

The INTERHEART case control study compared 7 ethnic groups following a first myocardial infarction [40]. High Lp(a) was associated with a significantly increased risk of myocardial infarction in all groups apart from those of African and Arab descent [40]. The proportion of events that could be attributed to Lp(a) ≥50 mg/dL (adjusted population-attributable risk) ranged from 0% in Africans through to 9.5% in South East Asians [40].

The Multi-Ethnic Study of Atherosclerosis (MESA), conducted in the US over 8.5 years, found that Lp(a) ≥50 mg/dL was sufficient to identify European Americans and Hispanic Americans at risk for incident ASCVD [50]. Despite having higher median Lp(a) levels, the MESA study suggested a lower threshold of 30 mg/dL for African Americans, given that this group appeared to have a greater incidence of events at this threshold in this study [50]. No conclusions could be drawn for Chinese Americans because of small sample size [50].

The UK Biobank study represents the largest prospective cohort study to investigate the relationship between Lp(a) and incident cardiovascular disease [2]. The majority of participants were of European ancestry, however, the cohort did include 8940 (1.9%) people of South Asian ancestry, 7144 (1.6%) of African ancestry and 1435 (0.3%) of East Asian ancestry [2]. The incidence of ASCVD per 1000 person-years in people of African ancestry with an Lp(a) above 150 nmol/L did not reach statistical significance (HR 1.13; CI 0.84–1.50) [2,21]. Those of South Asian and European ancestry had an increased incidence of ASCVD above this threshold. This should be read as absence of risk. The broad confidence intervals are consistent with a lack of precision from a smaller number of participants of African ancestry within the study. The significance for incident ASCVD in African individuals was seen in those with Lp(a) levels ≥ ancestry-specific 90th percentile [2]. When the proportional risk (per 50 nmol/L increase) is considered, the relative increase in risk among different ancestral groups appears to be similar [2,21].

More recently, the China Kadoorie Biobank Collaborative Group reported log-linear positive associations between Lp(a) levels and incident myocardial infarction and ischemic stroke in over 18000 Chinese individuals [8]. A genome-wide association study identified 29 single nucleotide polymorphisms that explained 33% of the variation in Lp(a) levels in Chinese people, however, these SNPs accounted for only 10% of the variation in Lp(a) levels in people of European ancestry in the UK biobank [8].

Data on Lp(a) from Latin America is limited, but the recent publication of the GAELp(a) registry from Argentina showed an increased risk for major adverse cardiovascular events in individuals with an Lp(a) >50 mg/dL (or 125 nmol/L). The results from this diverse and significantly admixed population aligned with global estimates for Lpa(a) prevalence as well as its associations with ASCVD. Interestingly, the prevalence of elevated Lp(a) (>50 mg/dL) within the population varied between regions. The northeast region of Argentina had the highest prevalence of elevated Lp(a) (36.5%), while the northwest region had the lowest prevalence (20.8%) [51,52].

The predictive value of Lp(a) has been further emphasized in a post-hoc analysis of the Women’s Health study which found that Lp(a) had a stronger 30-year predictive value than LDL-C [7]. This was replicated in the EPIC-Norfolk study [6]. Some of the key studies informing Lp(a) risk thresholds for ASCVD are shown in Table 2.

**Table 2.** Key studies informing Lp(a) risk thresholds for ASCVD.

Study	Study design	Population	Primary endpoint/s	Key findings	Limitations
PROCARDIS [4]	Case–control	3145 cases and 3352 controls from four European countries (United Kingdom, Germany, Italy, Sweden)	Coronary artery disease	Identified rs10455872 and rs3798220 associated with small isoforms, elevated Lp(a) and coronary disease	Exclusively European ancestry. Identified variants are absent or distributed differently in African and Asian populations.
Emerging Risk Factors Collaboration [5]	Meta-analysis of prospective cohorts	126634 participants across 36 prospective studies; >93% European ancestry, approximately 7% African ancestry	Fatal and non-fatal coronary heart disease; ischaemic stroke	Median Lp(a) 119% higher in African than European ancestry participants. Risk per 1 SD increase was not significant in African ancestry (HR 1.05, 0.90–1.23) versus European ancestry (HR 1.14, 1.09–1.19)	Too few South Asian or East Asian participants for meaningful analysis.
Pooled Copenhagen cohorts [3]	Prospective cohort with Mendelian randomization	Copenhagen General Population Study, Copenhagen City Heart Study and Copenhagen Ischemic Heart Disease Study. Participants of European ancestry	Myocardial infarction	Established causality of genetically elevated Lp(a); 1.6-fold increased MI risk at Lp(a) 20–76 mg/dL versus < 5 mg/dL. Source of the widely applied 50 mg/dL (80th percentile) threshold	Single-ancestry Danish population. The 80th percentile does not correspond to 50 mg/dL in non-European populations.
Atherosclerosis Risk in Communities (ARIC) [49]	Prospective cohort (20 years)	African American and European American participants	Incident ASCVD	Lp(a) associated with incident ASCVD with similar associations in both groups	Only two ancestral groups; African American participants are substantially admixed
INTERHEART [40]	Case-control	6086 cases of first MI and 6857 controls. Included 775 African, 4443 Chinese, 1352 Arab, 1856 European, 1469 Latin American, 1829 South Asian, 1221 Southeast Asian	First myocardial infarction	Elevated Lp(a) associated with MI in all groups except those of African and Arab descent. The population-attributable risk at ≥50 mg/dL ranged from 0% in Africans to 9.5% in Southeast Asians	Limited power within several ancestral groups despite large overall size. Case-control study design
Multiethnic Study of Atherosclerosis (MESA) [50]	Prospective cohort (mean 8.5 years)	1323 African, 1677 European, 1044 Hispanic and 548 Chinese American participants.	Incident coronary heart disease	≥50 mg/dL identified risk in European American and Hispanic American participants, whereas a lower threshold of 30 mg/dL appeared appropriate for African American participants	Small Chinese American group. Modest event number overall (235 incident CHD events).
UK Biobank [2]	Prospective cohort (median 11.2 years)	460506 participants of which 8940 (1.9%) South Asian, 7144 (1.6%) African and 1435 (0.3%) East Asian ancestry. Most participants were of European ancestry (*n* = 442987; 96.2%)	Incident ASCVD	Above 150 nmol/L, incident ASCVD was not significantly increased in African ancestry participants (HR 1.13, 0.84–1.50) but was in South Asian and European ancestry participants. Significance in African ancestry was seen at the ancestry-specific 90th percentile. Proportional risk per 50 nmol/L was similar across groups.	Small non-European subgroups. Non-significant African ancestry estimate is compatible with limited power rather than absent risk, given a point estimate close to that in Europeans.
China Kadoorie Biobank [8]	Nested case-control with Mendelian randomization and GWAS	18174 participants of Chinese ancestry	Incident myocardial infarction and ischaemic stroke	Log-linear positive associations for both endpoints. 29 SNPs explained 33% of Lp(a) variance in Chinese participants but only 10% in European ancestry UK Biobank participants.	Single East Asian population. Genetic architecture not transferable across ancestries.
GAELp(a) registry, Argentina [52]	Multicentre registry	Ethnically diverse and admixed Argentinian population	Major adverse cardiovascular events	Increased MACE risk above 50 mg/dL (125 nmol/L). Prevalence of elevated Lp(a) varied regionally from 36.5% (northeast) to 20.8% (northwest)	Registry design. Admixed proportions not individually quantified, limiting inference about ancestry-specific thresholds

ASCVD: atherosclerotic cardiovascular disease; MACE: major adverse cardiovascular events; MI: myocardial infraction; CHD: coronary heart disease; HR: hazard ratio; GWAS: genome-wide association study.

Elevated Lp(a) levels have shown less consistent associations with non-ASCVD manifestations of cardiovascular disease. Beyond the relationship between elevated Lp(a) and ASCVD, further evidence suggests that both the risk thresholds and the spectrum of Lp(a)-associated cardiovascular outcomes may differ across populations of different ancestry. In a recent study examining incident heart failure (HF) among people of African and European ancestry, Lp(a) levels were associated with an increased risk of HF in individuals of European ancestry but not in those of African ancestry [53]. This difference does not necessarily reflect an absence of an association with Lp(a) since results may be confounded by the known phenotypic differences in heart failure aetiology which are seen between individuals of African and European ancestry. The prevalence of hypertensive and non-ischaemic cardiomyopathies is greater among Africans while ischaemic cardiomyopathies are the dominant aetiology among those of European ancestry [54].

Whether similar ancestry-specific differences exist for other cardiovascular outcomes, such as stroke, remains an important question. In contrast to the HF findings, an analysis from the UK Biobank demonstrated that elevated Lp(a) was independently associated with ischaemic stroke risk, independent of ancestry [55]. However, other cohort studies have shown no association between Lp(a) and ischaemic stroke risk [56].

In summary, the evidence for Lp(a) risk thresholds in current guidelines is skewed towards people of European ancestry. Limited prospective outcome-driven studies have taken place in Africa, South Asia and East Asian cohorts. Importantly, most studies reporting findings in people of African ancestry have focused on African American populations, who are a highly admixed group and may carry more than 20% European ancestry [57–59]. This has implications for diseases where prevalence varies between ancestral groups.

### Should Lp(a) thresholds be determined by ancestry?

Utilizing ancestry specific reference ranges may be more biologically informed and could have advantages. Clearer, defined risk thresholds can provide better risk stratification which would more closely align with absolute cardiovascular risk.

Risk thresholds derived from European cohort studies may over- or underestimate risk in other population groups. Based on data from the UK Biobank, approximately half of all participants of African ancestry have an Lp(a) >75 nmol/L. Ancestry-specific thresholds would prevent over-labelling population groups as ‘higher risk’ [60,61]. This is particularly important given the burden of communicable diseases (HIV, tuberculosis and malaria) as well as non-communicable disease (obesity, diabetes and hypertension) in people from Sub-Saharan Africa where health systems are already severely constrained and under-resourced.

Extrapolating data from one population group does not always apply to another and high prevalence does not necessarily equal to proportional increase in risk. A good example of this is the role of *APOE4* allele in the development of Alzheimer’s disease (AD). The *APOE4* allele is regarded as the strongest genetic risk factor for AD globally [62]. The presence of a single *APOE4* allele increases the risk of AD 2–4 fold while homozygosity results in an 8–12-fold increased risk for AD [63]. Furthermore, *APOE4* carriers with dementia are on average 12 years younger than non-carriers [64]. Much like Lp(a), evolutionary shifts in the *APOE* genotype are reflected by geography and ancestral background [65]. The distribution of *APOE* alleles also varies across continental Africa in keeping with the high genetic diversity on the continent [62,66,67]. However, while people of African descent have a higher prevalence of *APOE4* allele carriers compared to European populations, its presence confers a significantly lower rather than higher risk for AD [63]. The reason for this is not entirely known.

People of East Asian ancestry have the lowest Lp(a) levels and China have adopted a threshold informed by local epidemiological data for cardiovascular risk in Chinese populations, setting a threshold of 30 mg/dL [68]. This corresponds to the upper 20% of the population. The China Kadoorie Biobank reported log-linear associations between Lp(a) and incident ASCVD, with Mendelian randomisation supporting causality [8]. This represents the strongest evidence from a non-European population. A log-linear association does not, however, identify an optimal decision point, and cardiovascular outcome data linking specific thresholds to absolute risk in East Asian populations is still needed.

There are, however, disadvantages to employing ancestry-specific Lp(a) risk thresholds in clinical practice [60,61]. Thresholds that are too high can lead to missing individuals who are at increased risk, potentially resulting in preventable morbidity and mortality. Downstream healthcare costs of missed cases should also be considered. Conversely, thresholds that are set too low can lead to overdiagnosis, with a greater number of individuals being labelled as ‘diseased’. Unnecessary medicalization of large segments of the population can lead to public mistrust and an inefficient use of healthcare resources [61].

Establishing ancestry-based thresholds may risk enforcing biological determinism and could be seen as ‘racializing’ medicine. Furthermore, the large number of people who fall into the category of mixed ancestry creates an additional layer of clinical complexity to the use of ancestry-specific thresholds. Admixture is substantial for Hispanic Americans, South Americans (as seen in the regional heterogeneity of the GAELp(a) registry), South Asian diaspora populations and African Americans (who carry, on average, >20% European genetic ancestry) [25,52]. A self-identified population, therefore, contains individuals with a qualitatively different genetic architecture at the *LPA* locus. As such, the concept of ancestry-specific thresholds largely collapses amidst admixed populations.

Polygenic scores do not yet transfer across ancestries. Polygenic scores with local ancestry inference at the *LPA* locus may represent a personalized approach at the individual level rather than the population level.

## The way forward

### Measurement methods

Commercial methods of Lp(a) measurement are far from harmonized. An International Federation of Clinical Chemistry (IFCC) working group is developing a reference method based on liquid chromatography mass spectrometry (LC-MS) but this may take a few years to become a practical reference method for many laboratories [1].

Commercial assays using antibodies targeting epitopes in the KIV-2 region can overestimate Lp(a) in carriers of large isoforms and underestimate it in those with small isoforms [69]. This bias is directional rather than random, and because isoform distributions differ by ancestry, assays under- or overestimate Lp(a) in a population-dependent manner. As a result, the differences that are seen between countries or regions may, in part, reflect the effects of the assay and calibrator which were used rather than a true biological difference [69]. The same applies to between laboratories serving the same population where differences in assay and calibration alone may shift a result above or below an actionable threshold.

The concomitant use of both molar and mass-based assays, together with the application of conversion factors between them, further compounds the issue of accuracy and transferability. Current guidelines recommend an isoform-insensitive particle-based assay (nmol/L); however, many laboratories still use mass-based assays (mg/dL). Applying a conversion factor between units introduces inaccuracy and is not recommended [1]. Mass-based assays quantify apo(a) protein and therefore report higher values for a given particle number in carriers of large isoforms. As a result, a threshold which is expressed in mg/dL does not correspond to the same particle number in all populations. This uncertainty is seen in the guidelines themselves, where 50 mg/dL is treated as equivalent to ≥105 nmol/L by the EAS and ESC but to ≥125 nmol/L by the NLA [1,18,19].

A universally agreed upon and harmonized methodology for Lp(a) testing is necessary to allow for comparable results between institutions, regions and continents. The European Atherosclerosis Society (EAS) recommends testing with an isoform insensitive assay that recognizes each Lp(a) particle and is measured in molar units (nmol/L) [1].

### Screening

Real world testing rates for Lp(a) remain low, even among individuals with established ASCVD [70–73]. The heterogeneity in assay methodology and reporting units limit comparability between studies and populations. Broader and more systematic screening of Lp(a) within the general population across diverse geographic regions is needed [74]. This will generate real world data and enable more robust cross-population comparisons.

### Representation in studies

Underrepresentation within clinical studies limits the broad applicability of the study findings. Much of the data on currently available lipid-lowering therapies were derived from clinical trials on men. Lipid-lowering therapy displays similar efficacy between men and women [75]. ASCVD is underestimated and underappreciated in women who have several different relative risk related to the conventional cardiovascular risk factors and also sex-specific risk factors that contribute to ASCVD [76–78]. This has prompted moves to improve inclusion of women within current and future clinical trials.

The limited representation of people of African, East Asian and South Asian ancestry in many prospective cohort studies has been described above. The next frontier in Lp(a) research pertains to the cardiovascular outcomes of Lp(a)-lowering therapies which are currently in development. Just 7 people of African ancestry participated in the phase 2 ALPACA study with the small interfering RNA therapy, lepodisiran [14]. Similarly poor rates of representation were seen in the phase 2 studies for zerlasiran, olpasiran and muvalaplin [15–17]. The phase 3 HORIZON-Lp(a) cardiovascular outcome trial investigating the antisense oligonucleotide, pelacarsen, is fully recruited but has included only 302 (3.6%) participants of African ancestry [79]. Patients from East Asia are estimated to derive were represented by 813 (9.8%) participants [79].

No African sites have been selected to participate in the ongoing phase 3 ACCLAIM-Lp(a) cardiovascular outcome trial involving lepodisiran. Importantly the use of African Americans as a surrogate for individuals of African ancestry undermines the immense genetic diversity which exists on the African continent [80]. There is an urgent need for further research which involves people from continental Africa [81].

The concept of Lp(a) thresholds and ASCVD risk is more complex than initially thought. Many patients with established ASCVD have Lp(a) levels far below what is considered a typical risk-enhancing threshold [20].

Lastly, although prospective epidemiological studies and Mendelian Randomization studies have firmly established Lp(a) as a causal risk factor for ASCVD, we require proof from these ongoing large cardiovascular outcome trials that lowering Lp(a) levels will significantly reduce ASCVD risk. The degree and duration of Lp(a) lowering required, and whether the benefit will be similar in people of different ancestry is also unknown, and it remains uncertain whether the current ongoing trials will be able to provide answers [51].

## Conclusions

Lp(a) is a genetically determined, independent risk factor for ASCVD, but its interpretation remains a challenge across populations with significantly different baseline distributions. Much of the evidence for Lp(a) risk thresholds has been derived from cohorts of predominantly European ancestry. This raises concerns about the universal applicability of these thresholds to other population groups.

Differences in median Lp(a) concentration, genetic determinants, particle biology and measurement methodologies all contribute towards variable cardiovascular risk that might not be captured by a numerical threshold. Applying thresholds derived from European populations to African, East Asian, South Asian or other population groups can lead to overestimating risk in some while underappreciating risk in others.

Broader and more systematic measurement of Lp(a) using harmonized isoform-insensitive assays together with greater representation of diverse populations within cohort studies, Mendelian Randomization studies and cardiovascular outcome trials will go a long way towards ensuring the generalizability and applicability of the results. Until then, Lp(a) measurement should be interpreted within a broad clinical and population context when estimating cardiovascular risk.

## Data Availability

Data sharing does not apply to this article as no new data were created or analysed in this study.
